# Superior Cluneal Nerve Entrapment as a Cause of Low Back Pain Refractory to Sacroiliac Joint Fusion: A Case Report

**DOI:** 10.7759/cureus.44271

**Published:** 2023-08-28

**Authors:** Jacob L Hostetter, Kamal Patel

**Affiliations:** 1 Anesthesiology, Lake Erie College of Osteopathic Medicine, Bradenton, USA; 2 Pain Management, NeuSpine Institute, Wesley Chapel, USA

**Keywords:** sacroiliac joint, iliac crest, opioids, superior cluneal nerve entrapment, low back pain

## Abstract

Low back pain (LBP) is a common complaint that can be nonspecific. Superior cluneal nerve entrapment should be included in the differential for LBP because, without a precise diagnosis, treatment may be less effective. A 61-year-old female with a history of chronic LBP and sacroiliac (SI) pain requiring opioids for pain control presented with minimal relief following SI joint fusion. Physical exam showed tenderness over the iliac crest with burning, radicular pain into the buttock. The patient received a superior cluneal nerve injection of local anesthetic that provided 100% pain relief for 72 hours without the use of opioids and no complaints of burning or radicular pain. This confirmed the diagnosis of superior cluneal nerve entrapment syndrome causing superior cluneal neuralgia. Superior cluneal nerve entrapment syndrome should be considered when evaluating causes of LBP to avoid unnecessary procedures and reduce the use of opioids.

## Introduction

Low back pain (LBP) is a very common complaint in medicine and is often nonspecific [1]. It is seen to be a nonspecific complaint in 85% of people [2]. This can lead to expensive imaging, diagnostic tests, and treatments to make a diagnosis and attempt to provide pain relief. LBP costs over $100 billion per year in the United States [3]. One cause of LBP that often localizes to the iliac crest and the buttock region is superior cluneal nerve entrapment. The superior cluneal nerves are sensory nerves that consist of cutaneous branches of the posterior rami of the T11-L5 nerve roots [4]. These nerves run from superior-medial to inferior-lateral and penetrate the thoracolumbar fascia at the iliac crest. The medial branch is the most commonly entrapped and makes this penetration about 3-4 cm from the midline [5]. Clinical signs pointing to a diagnosis of superior cluneal nerve entrapment include pain exacerbated by lumbar extension, lateral flexion, rotation, prolonged standing, pain, and numbness radiating down the leg. A positive response to anesthetic injection at the site of nerve penetration at the posterior iliac crest is useful in confirming the diagnosis [6].

The diagnosis of superior cluneal nerve entrapment is made by physical examination findings, pain in the region supplied by the superior cluneal nerves, and a therapeutic response to local anesthetic injection. These are all important in differentiating from other causes of LBP. Localization of pain at the posterior superior iliac spine with a positive Faber’s test producing pain localizing to the lower back may indicate sacroiliac (SI) joint pathology [6]. SI joint pain can be seen together with superior cluneal nerve entrapment, but it is important to differentiate the two. This case represents a patient with LBP uncontrolled by SI joint fusion surgery and opioids who received 100% pain relief with local anesthetic, suggesting a diagnosis of superior cluneal nerve entrapment syndrome. Informed consent was obtained from the patient for the publication of this case report.

## Case presentation

The patient is a 61-year-old Caucasian female homemaker who presented with constant LBP that started just over one year ago that she described as aching, stabbing, and rated an 8/10 using the numerical rating scale (NRS), most significant over the left iliac crest. She reported burning, radicular pain in her left buttock, but denied urinary problems, fever, and chills. She takes oxycodone-acetaminophen 5-325 mg three times daily as needed with some relief of pain. She reported worsening pain with physical activity such as completing chores around the house. Her pain is aggravated by standing, walking, extension, and rotation. Her pain is alleviated by pain medication and rest. Previous trials of home physical therapy and over-the-counter pain medication have failed to alleviate her pain.

A physical exam revealed a patient in no acute distress with tenderness to palpation over the left iliac crest. Vital signs showed a temperature of 36.7 C, body mass index of 32.57 kg/m2, heart rate of 98 bpm, and blood pressure of 129/79 mmHg. Light touch, pinprick, vibration, and position sensation were intact. Faber’s test was positive on the left. The patient was alert and oriented with a euthymic mood and had normal judgment and a full range of affect.

Past medical history is significant for anxiety, depression, asthma, type 2 diabetes, chronic obstructive pulmonary disease, headaches, and osteoarthritis. Past surgical history is significant for a left SI joint fusion one year ago with minimal benefit. She reported no significant family history of medical conditions and does not use alcohol or tobacco. She has significant family stress factors with the deaths of multiple family members. She reported this contributing to her underlying depression which augments her pain. Her depression is currently well-controlled on amitriptyline 100 mg daily and duloxetine 60 mg daily.

**Figure 1:**
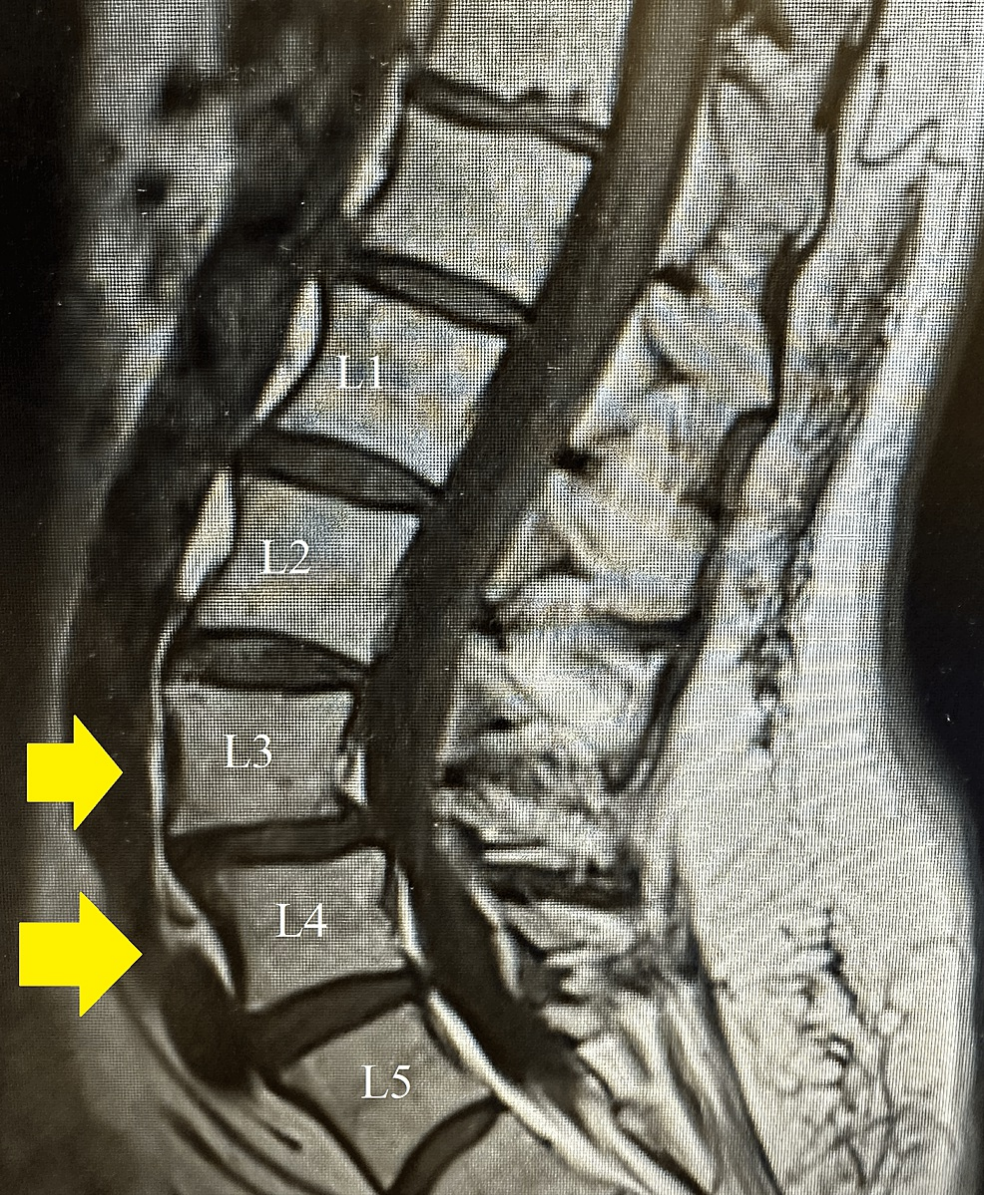
Lumbar MRI The yellow arrows identify the L3 and L4 vertebrae exhibiting grade 1 spondylolisthesis. A grade 1 spondylolisthesis is slippage of less than 25% of the superior vertebral body relative to the vertebral body below

Previous lumbar MRI showed L3 and L4 grade 1 spondylolisthesis with foraminal stenosis and facet arthropathy (Figure 1). She previously received bilateral SI joint injections with local anesthetic and left L4 and L5 epidural steroid injections. She reported pain relief with right-sided SI injections but still had pain on her left side. She reported about 80% relief of LBP with the epidural steroid injections.

This presentation suggested the diagnosis of superior cluneal nerve entrapment syndrome. She received a left superior cluneal nerve injection of local anesthetic which provided 100% pain relief for 72 hours, confirming the diagnosis. The patient verbalized complete relief of her pain and satisfaction with the procedure. This positive response made the patient a great candidate for superior cluneal nerve radiofrequency ablation. The patient stated she was not interested in pursuing therapy with a peripheral nerve stimulator. Definitive treatment was provided with left superior cluneal nerve radiofrequency ablation which decreased a pre-procedure NRS pain level of 8/10 to a post-procedure pain level of 0/10. Future follow-up will be directed at discussing long-term outcomes and reassessing the requirement for opioid pain medication.

## Discussion

The reported incidence of superior cluneal nerve entrapment is between 1.6% and 14% in patients with LBP [7]. It is important to differentiate between the causes of LBP, in part to avoid potentially unnecessary or excessive imaging and to achieve more effective treatment and better outcomes for patients. A study published in 2009 found that lumbar imaging for LBP without indications of serious underlying conditions does not improve clinical outcomes and recommended clinicians refrain from routine, immediate lumbar imaging in patients with acute or subacute LBP without features suggesting a serious underlying condition [8]. A thorough history and physical examination are vital parts of determining the proper treatment for LBP.

The most common cause of LBP is nonspecific pain that is often musculoskeletal [9]. The majority of patients with LBP improve within the first several weeks. Initial therapy for acute back pain includes the application of heat, massage, spinal manipulation, exercise, physical therapy, or nonsteroidal anti-inflammatory drugs [10]. Failure to improve after four to six weeks requires further evaluation.

This case shows that LBP is often multifactorial. This patient has a history of L3 and L4 grade 1 spondylolisthesis and lumbar spinal stenosis confirmed by MRI. Symptoms associated with this condition include pain, numbness, or tingling that may be exacerbated by standing or walking [11]. Patients with chronic neuropathic pain are initially treated with either a tricyclic antidepressant, serotonin-norepinephrine reuptake inhibitors, or antiseizure medication, and this choice can be dependent on comorbid conditions [12]. This patient with comorbid depression was treated with a combination of amitriptyline and duloxetine.

It is also important to differentiate LBP from SI joint pain. These conditions can often be seen together, and the prevalence of SI joint dysfunction among patients with LBP is estimated to be 15% to 30% [13]. SI joint pain localizes to a 3 cm by 10 cm area inferior to the ipsilateral posterior superior iliac spine. It can often be difficult to truly differentiate SI joint pain from LBP because the referred pain of SI joint dysfunction typically extends in the L5-S1 nerve distribution to the buttocks, groin, posterior thigh, and lower leg [13]. This patient was treated previously for SI joint pain with an injection of local anesthetic and a left-sided SI fusion procedure that did not provide adequate relief of pain. This is an example of when it may be necessary to consider other causes of LBP such as superior cluneal nerve entrapment.

The superior cluneal nerves are sensory nerves that consist of cutaneous branches of the posterior rami of the T11-L5 nerve roots [4]. These nerves run from superior-medial to inferior-lateral and penetrate the thoracolumbar fascia at the iliac crest. The medial branch is the most commonly entrapped and makes this penetration about 3-4 cm from the midline [5]. One treatment that can be effective for superior cluneal nerve pain that responds to diagnostic injection of local anesthetic is radiofrequency ablation. The evidence supports radiofrequency ablation, a procedure using heat to interrupt pain signals, as an efficacious treatment for lumbar facet joint and SI joint pain [14]. More research is needed on its effectiveness for superior cluneal nerve entrapment in particular.

One very common treatment for LBP is the use of opioid analgesics. Data shows that opioids are the most commonly prescribed class of drugs for back pain, and more than half of regular opioid users report back pain [15]. Increased opioid use has been associated with increased drug abuse and deaths from overdose [16]. Opioids have a high addictive potential and are associated with adverse effects such as sedation, dizziness, nausea, vomiting, constipation, physical dependence, tolerance, and respiratory depression [17]. It is important to recognize the presentation of superior cluneal nerve entrapment as a cause of LBP, in part to avoid risks associated with excessive opioid prescription.

## Conclusions

In conclusion, superior cluneal nerve entrapment is a cause of LBP that can be treated effectively when accurately diagnosed. Pain associated with this condition is located lying over the posterior iliac crest. A therapeutic reduction in pain in response to local anesthetic injection confirms the diagnosis. Early recognition of this condition can aid in reducing higher risk and more invasive treatments of LBP including opioid prescription and surgery. Physicians should be aware of this condition as a cause of LBP to improve patient outcomes and reduce unnecessary treatment.
